# Relationship between disease severity and folate status of children with sickle cell anaemia in Enugu, South East Nigeria

**DOI:** 10.4314/ahs.v21i2.35

**Published:** 2021-06

**Authors:** Uchenna C Nnajekwu, Chukwubike O Nnajekwu, Vivian O Onukwuli, Ndubuisi A Uwaezuoke, Osita U Ezenwosu, Anthony N Ikefuna, Ifeoma J Emodi

**Affiliations:** 1 Department of Paediatrics, University of Nigeria Teaching Hospital, Enugu, Nigeria; 2 Department of Paediatrics, College of Medicine, University Of Nigeria, Enugu Campus

**Keywords:** Sickle cell anaemia, disease severity, folate status, children, Enugu

## Abstract

**Background:**

Repeated crises in children with sickle cell anaemia (SCA), which is a manifestation of disease severity, results in depletion of their minimal tissue folate stores, with higher likelihood of folate deficiency. The study aimed to determine the relationship between disease severity and the folate status of children with SCA attending University of Nigeria Teaching Hospital (UNTH), Enugu.

**Methods:**

This was a hospital based, cross-sectional study conducted between September 2018 and March 2019. One hundred participants were recruited, consisting of 50 children having sickle cell crisis and 50 age and gender matched haemoglobin AA genotype controls. Relevant information was documented using a pretested questionnaire. Sickle cell severity score was determined using frequency of crisis, admissions and transfusions in the preceding one year, degree of liver and splenic enlargement, life-time cummulative frequency of specific complications of SCA, leucocyte count and haematocrit.

**Results:**

Folate deficiency was observed in eight percent of the subjects and none of the controls. The difference was not significant (Fisher's exact = 4.167, p=0.117). The odds of being folate deficient was 8.5 times more likely during anaemic crisis than in vaso-occlusive crisis, though not significant (95% C.I 0.05 – 89.750, p = 0.075). The mean SCA severity score was 8.06 ± 3.64, signifying a moderate SCA severity in the study population. There was a no relationship between folate status and severity of SCA (Fisher's exact = 0.054, p = 0.949)

**Conclusion:**

Folate status in children with SCA is not affected by their disease severity. Therefore, there may be no need for additional folate supplementation with increasing severity of sickle cell anaemia.

## Introduction

Sickle cell anaemia is the commonest genetic disease worldwide.^1^ The highest population of people with sickle cell anaemia is seen among those of African origin.^2^ About three percent of the Nigerian population of over 160 million have sickle cell anaemia.^1^

The severity of the symptoms of SCA vary from person to person, and ranges from mild to life-threatening. The severity of SCA can be assessed using clinical and/or laboratory parameters, though there is no accepted standard method for determining the severity of sickle cell anaemia. Prompt and proper assessment of disease severity by the managing physician makes it easier to identify high risk patients who may benefit from chronic transfusion programme, exchange blood transfusion, hydroxyurea, and bone marrow transplantation. Folate is necessary for the conversion of homocysteine to methionine.^3^ Deficiency of folate leads to accumulation of homocysteine, which is a highly reactive sulphur-containing amino acid known to cause injury and dysfunction of the endothelium of blood vessels, and thrombin formation.^4^ Therefore, deficiency of folate and resultant hyperhomocystinaemia could increase the frequency of thrombotic events and painful episodes in children with sickle cell anaemia.^3^ Children who have frequent crises have lower steady state haemoglobin due to increased haemolysis, and higher folate demand.^5^ Homocysteine concentration is an indirect measure of folate status, but is not specific for folate since it can be influenced by factors such as renal dysfunction, cobalamine deficiency, and levels of other micronutrients.^6^

There has been documentation of significant association between severity of SCA and hyperhomocystinaemia,^7^–^8^ but literature on the relationship between folate status and severity of SCA is lacking. The aim of this study was to determine the relationship, if any, between the severity of SCA and folate status. Findings from this study will guide appropriate decision making in the management of children with SCA, and reduce the morbidity and mortality associated with the disease.

## Methods

The study was hospital based and cross-sectional, conducted at the University of Nigeria Teaching Hospital, Enugu, between September 2018 and March 2019. Fifty children with SCA aged two to seventeen years, who were receiving five milligrammes of folate as a daily supplement were recruited during crisis. The controls were fifty children age and gender matched apparently healthy children, who had haemoglobin AA genotype following haemoglobin electrophoresis done at recruitment.

Exclusion criteria included children who were receiving medications which affect folate levels such as antacids, H2-receptor blockers, carbamazepine, phenytoin, and methotrexate, also excluded were children who were transfused less than three months before recruitment, and children who had diarrhoea that lasted for more than fourteen days in the preceding one month. Ethical approval was obtained from the Health Research Ethics Committee of UNTH. Informed consent and assent were obtained from care-givers and study participants as appropriate.

The clinicolaboratory method of determination of sickle cell severity score proposed by Adegoke and Kuti^9^ was used for the study. This scoring system has been validated for use in Nigeria and Africa.^9^,^10^ Whole blood samples were taken under standard laboratory precautions for full blood count and red cell folate estimation with ECLIA method using the automated Mindray BC-5300 and Roche Cobas e411 equipment respectively. Red cell status was categorized as follows: folate levels less than 140 ng/ml were regarded as low, while levels between 140 and 620 ng/ml were categorized as normal folate status.

### Calculation of sickle cell severity score

Frequencies of painful crisis, hospitalization and blood transfusions in the preceding year were obtained from caregivers or subjects in the proforma. Each of these parameters was scored from zero to three. The subject was assigned a score of one each for one painful crisis, transfusion, hospital admission, two each for two or three painful crises, transfusions, and hospital admissions, and three each for more than three painful episodes, transfusions, and hospital admissions in the preceding year.^9^

Information regarding complications of sickle cell anaemia was obtained from the patient's medical records. Cerebrovascular accident was scored five, acute chest syndrome and pneumococcal meningitis scored three, avascular necrosis of the hip was given a score of two, while gall stones, chronic leg ulcers, osteomyelitis, and priapism were assigned a score of one each, irrespective of how many times these complications occurred.^9^

Subjects were examined by the researcher to determine the degree of liver and splenic enlargement. A score of zero in both parameters signified a liver enlargement less than two centimetres and a splenic enlargement less than five centimetres, one in each of the parameters signified a liver enlargement of two to five centimetres, and a splenic enlargement of five to ten centimetres, while a score of two in each of the parameters signified a liver enlargement greater than five centimetres, and a splenic enlargement of greater than ten centimetres.^9^

Haematocrit and total leucocyte count for each subject were extracted from the full blood count result and scores were assigned to each of the parameters, according to severity. Each of the parameters was scored from zero to two. Haematocrit of ≥ 24% was scored zero, 18 to 23% scored one, while < 18% was scored two.^9^ For total leucocyte count, a score of zero was assigned when the total white blood cell count was < 11 x 10^9^/µL, one was assigned when the count was between 11 and 15 x 10^9^/µL, and two assigned when the count was > 15 x 10^9^/µL.^9^ The total sickle cell severity score was obtained by adding the scores obtained from the different subgroups (history, physical examination, laboratory parameters).

The total severity score ranged from zero to thirty four, and stratified as: mild disease (total score of zero to seven), moderate disease (total score of eight to seventeen), and severe disease (total score of more than seventeen).^9^

Classification of subjects based on the type of crisis was done with their presenting complaints. Pain in any part of the body, irrespective of the severity, was grouped into the vaso-occlusive crisis (VOC) category, while worsening yellowness of the eyes, haematuria, severe paleness of the body, progressive hepatosplenomegaly, or features of anaemic heart failure were grouped into the anaemic crisis category.

Data analysis was done using the Statistical Package for Social Sciences version 20 (SPSS 20 Chicago). Categorical data were summarized as frequencies and percentages. Fisher's exact test was used to determine the proportion of subjects and controls with folate deficiency and the relationship between severity ofCA and the folate status. Odd's ratio was used to determine the likelihood of folate deficiency in anaemic and vaso occlusive crises. Probability (p) values less than 0.05 were regarded as significant.

## Results

Fifty children with SCA, made up of 29 males and 21 females, (M: F- 1.38:1) were studied. The ages of subjects and controls ranged from 2 years to 17 years, (mean 10.58 ± 3.73 years). A greater proportion (48%) of the study population were aged between 7 and 11 years.

Table 1 shows the distribution of the socio-demographic characteristics of the subjects and controls.

**Table 1:** Socio-demographic characteristics of study participants

Sociodemographic characteristic	Frequency (%)		Test statistic	df	p-value
				
	Control	Subjects			
Gender					
Male	29(58.0)	29(58.0)	0.000 a	1	1.000
Female	21(42.0)	21(42.0)			
Total	50(100.0)	50(100.0)			
Age (years)					
2 to 6	6(12.0)	6(12.0)	0.125^b^	3	1.000
7 to 11	24(48.0)	24(48.0)			
12 to 16	16(32.0)	16(32.0)			
>16	4(8.0)	4(8.0)			
Total	50(100.0)	50(100.0)			

a Fisher's exact test

b chi square

df degree of freedom.

### Severity of sickle cell anaemia in the subjects

The severity scores of the study population ranged from 0 to 19, with a mean score of 8.06 ± 3.64. Majority (52%) of the children studied had moderate disease. Folate status of the study population

Table 2 describes the folate status of the study population. Folate levels were below normal limits in 8% of the subjects during crisis. None of the controls had folate deficiency. The difference in proportion of subjects and controls with folate deficiency was not significant (Fisher's exact level of significance = 4.167, p = 0.117).

**Table 2:** Folate status of the study population

Study group	Folate Status N(%)		N (%)	Fisher's	df	p-value
					
	Low	Normal				
Subjects	4 (8.0)	46 (92.0)	50(100.0)	4.167	1	0.117
Control	0 (0.0)	50(100.0)	50(100.0)			

Fisher's: Fisher's exact level of significance, df degree of freedom

### Folate status during different types of crises

Table 3 describes the folate status of the subjects in different types of crises. Of the fifteen subjects having anaemic crisis, three (20%) had folate deficiency, while one (2.9%) out ofthe thirty five subjects with VOC had folate deficiency. The odds of being folate deficient was 8.5 times higher during anaemic crisis than in VOC. This was however, not significant (95% C.I 0.805 – 89.750, p = 0.075).

**Table 3:** Folate status of the subjects in different types of crises

Type of crisis	Folate status N(%)		Total N(%)	Fisher's	df	OR	95% C.I	p-value
						
	Low	Normal						
Anaemic	3 (20.0)	12 (80.0)	15 (100.0)	4.193	1	8.500	0.805 – 89.750	0.075
VOC	1 (2.9)	34 (97.1)	35 (100.0)					

Fisher's: Fisher's exact level of significance, df degree of freedom, OR: Odd's ratio, C.1: Confidence Interval

### Relationship between severity of sickle cell anaemia and folate status

Table 4 shows the relationship between the severity of sickle cell anaemia and folate status. There was no significant relationship between the severity of SCA and the folate status (Fisher's exact level of significance = 0.054, p = 0.949).

**Table 4:** Relationship between severity of sickle cell anaemia and folate status

Severity of SCA	Folate status		Total N(%)	Fisher's	df	p-value
					
	Low N (%)	Normal N (%)				
Mild	2 (8.7)	21 (91.3)	23(100.0)	0.054	2	0.949
Moderate	2 (7.7)	24 (92.3)	26 (100.0)			
Severe	0 (0.0)	1 (100.0)	1 (100.0)			

df: degree of freedom, Fisher's: Fisher's exact level of significance.

## Discussion

Eight percent of the subjects had folate deficiency during sickle cell crisis. The prevalence of folate deficiency during crisis compares favourably with findings documented by Liu^11^ and Watson-Williams, 12 but varies considerably with the report of Kennedy et al 13 who observed a higher prevalence rate of 15% in children with SCA. The odds of being folate deficient was 8.5 times higher in anaemic crisis compared to VOC. This was however not significant, probably due to the small sample size studied. Akinsulie 14 in Lagos also found a higher prevalence of folate deficiency in subjects with anaemic crisis. The finding of more subjects with folate deficiency during anaemic crisis could be explained by the severe haemolysis which characterizes anaemic crisis. This leads to increased demand of folate for deoxyribonucleic acid (DNA) synthesis and production of new erythrocytes to replace the destroyed ones.^15^ Depletion of the folate stores could increase the risk of folate deficiency. The higher prevalence of folate deficiency observed during anaemic crisis despite folate supplementation suggests that there may be need for increased dose of folate during anaemic crisis, in order to replenish tissue stores.

None of the controls had folate deficiency. This was similar to the findings observed by Liu.^11^ In contrast, other studies reported high prevalence of folate deficiency in controls.^14^,^15^ The absence of folate deficiency in the controls could be explained by the fact that the controls had haemoglobin genotype AA, with half-life of 120 days, and the rate of destruction of the effete erythrocytes in children with AA genotype is not severe enough to deplete the folate stores and cause deficiency of folate.^16^

The mean SCA severity score was 8.06 ± 3.64, signifying moderate disease. This is consistent with earlier studies in Nigeria and some parts of Africa.^9^,^10^,^17^ On the other hand, Al-Saqladi et al 18 in Yemen observed that a higher proportion of subjects were in the severe disease category. The differences observed could be due to genetic variations of haplotypes in the different countries where the studies were carried out. The predominant haplotype in Nigeria and some parts of Africa is the Benin Haplotype (BEN) which has intermediate levels of fetal haemoglobin, and confers a moderate disease severity,^9^,^10^,^17^ whereas, the predominant haplotypes in Yemen are a combination of the African and Saudi-Indian haplotypes (ARAB) which confer disease severity ranging from mild to severe.^18^

There was no significant relationship between the severity of SCA and the folate status. In severe sickle cell anaemia the level of haemoglobin F which improves the outcome of the disease is low.^19^ Low haemoglobin F leads to increased sickling and expression of adhesive molecules such as vascular cell adhesion molecule-1 (VCAM-1), intercellular adhesion molecule-4 (ICAM-4), and basal cell adhesion molecule (BCAM).^16^ These molecules cause the erythrocytes to stick to themselves and the endothelium of the blood vessels.^16^ They could also cause adherence to the macrophages, with subsequent erythrophagocytosis and haemolysis. As a result of this, the stores of folate could be used up in a bid to replace the destroyed erythrocytes, leading to lower folate levels in severe disease.^16^ The observations from the study differed from this expectation. The lack of relationship between severity of SCA and the folate status could have been because of the small number of subjects, as only two subjects had severe disease, limiting statistical deduction. In addition, the lack of relationship could be explained by the routine folate supplementation in the subjects, as this could have obviated the effects of the crisis on the folate levels. There is a dearth of literature on the relationship between severity of SCA and folate status. Findings from the study show that disease severity had minimal effect on folate status and the dose of folate should be sustained irrespective of the degree of severity of SCA.

## Conclusion

Severity of SCA had no relationship with folate levels and there may be no need for additional folate supplementation with increasing severity of SCA.
